# Supplementary material for: Utilizing the ABC Transporter for Growth Factor Production by fleQ Deletion Mutant of Pseudomonas fluorescens
Source: Biomedicines. 2021 Jun 16;9(6):679. doi: 10.3390/biomedicines9060679 (PMC8234862; doi:10.3390/biomedicines9060679)
Supplement: Supplementary file 1 [file biomedicines-09-00679-s001.zip › biomedicines-1185930-supplementary.pdf]

biomedicines

Utilizing the ABC transporter for growth factor Production by *fleQ* Deletion Mutant of *Pseudomonas fluorescens*

Benedict Uy Fabia[1], Joshua Bingwa[1], Jiyeon Park[2], Nguyen Mihn Hieu[1], and Jung Hoon Ahn[1,2,*]

Supplementary material. Full sequences of the target proteins, in FASTA formats

> IGFI
MGPETLCGAELVDALQFVCGDRGFYFNKPTGYGSSSRRAPQTGIVDECCFRSCDLRRLEMYCAPLKPAKSA

> IGFII

MAYRPSETLCGGELVDTLQFVCGDRGFYFSRPASRVSRRSRGIVEECCFRSCDLALLETYCATPAKSE

> βNGF

MSSSHPIFHRGEFSVCDSVSVWVGDKTTATDIKGKEVMVLGEVNINNSVFKQYFFETKCRDPNPVDSGCRGIDSKHW
NSYCTTTHTFVKALTMDGKQAAWRFIRIDTACVCVLSRKAVRRA

> FGF1
MFNLPPGNYKKPKLLYCSNGGHFLRILPDGTVDGTRDRSDQHIQLQLSAESVGEVYIKSTETGQYLAMDTDGLLYGS
QTPNEECLFLERLEENHYNTYISKKHAEKNWFVGLKKNGSCKRGPRTHYGQKAILFLPLPVSSD

> TGFβ
MSRALDTNYCFSSTEKNCCVRQLYIDFRKDLGWKWIHEPKGYHANFCLGPCPYIWSLDTQYSKVLALYNQHNPGA
SAAPCCVPQALEPLPIVYYVGRKPKVEQLSNMIVRSCKCS

> TNFβ

MLPGVGLTPSAAQTARQHPKMHLAHSTLKPAAHLIGDPSKQNSLLWRANTDRAFLQDGFSLSNNSLLVPTSGIYFV
YSQVVFSGKAYSPKATSSPLYLAHEVQLFSSQYPFHVPLLSSQKMVYPGLQEPWLHSMYHGAAFQLTQGDQLSTHT
DGIPHLVLSPSTVFFGAFAL

> Negatively supercharged IGFI (IGFI(-))
MGPETLCGAELVDALQFVCGDRGFYFNEPTGYGSSSQRAPQTGIVDECCFRSCDLQQLEMYCAPLEPAKSA

> Negatively supercharged IGFII (IGFII(-))

MAYRPSETLCGGELVDTLQFVCGDRGFYFSEPASQVSQQSRGIVEECCFRSCDLALLETYCATPAKSE

> Negatively supercharged βNGF (βNGF(-))

MSSSHPIFHRGEFSVCDSVSVWVGDKTTATDIKGKEVMVLGEVNINNSVFKQYFFETKCRDPNPVDSGCRGIDSKHW
NSYCTTTHTFVKALTMDGEQAAWDFIDIDTACVCVLSEEAVEEA

> **Negatively supercharged FGFI (FGF1(-))**
MFNLPPGNYQQPKLLYCSNGGHFLRILPDGTVDGTRDDSDQHIQLQLSAESVGEVYIKSTETGQYLAMDTDGLLYGS
QTPNEECLFLERLEENHYNTYISQEHAEQNWFVGLKQNGSCKDGPRTHYGQKAILFLPLPVSSD

> Negatively supercharged TGFβ (TGFβ(-))
MSRALDTNYCFSSTEENCCVRQLYIDFREDLGWKWIHEPKGYHANFCLGPCPYIWSLDTQYSKVLALYNQHNPGAS
AAPCCVPQALEPLPIVYYVGRDPKVEQLSNMIVDSCKCS

> Negatively supercharged TNFβ (TNFβ(-))

MLPGVGLTPSAAQTAQQHPQMHLAHSTLKPAAHLIGDPSTQNSLLWRANTDRAFLQDGFSLSNNSLLVPTSGIYFV
YSQVVFSGEAYSPEATSSPLYLAHEVQLFSSQYPFHVPLLSSQKMVYPGLQEPWLHSMYHGAAFQLTQGDQLSTHTD
GIPHLVLSPSTVFFGAFAL
